# Early Infant Feeding Practices and Associations with Growth in Childhood

**DOI:** 10.3390/nu16050714

**Published:** 2024-02-29

**Authors:** Priscilla K. Clayton, Diane L. Putnick, Ian R. Trees, Akhgar Ghassabian, Jordan N. Tyris, Tzu-Chun Lin, Edwina H. Yeung

**Affiliations:** 1 Epidemiology Branch, Division of Population Health Research, *Eunice Kennedy Shriver* National Institute of Child Health and Human Development, National Institutes of Health, 6710B Rockledge Dr, Bethesda, MD 20817, USA; priscilla.clayton@nih.gov (P.K.C.); putnickd@mail.nih.gov (D.L.P.); ian.trees@nih.gov (I.R.T.); 2 Department of Pediatrics and Population Health, NYU Grossman School of Medicine, 550 First Avenue, New York, NY 10016, USA; akhgar.ghassabian@nyulangone.org; 3 Division of Hospital Medicine, Children’s National Hospital, 111 Michigan Avenue NW, Washington, DC 20010, USA; jbarger@childrensnational.org; 4 Glotech Inc., 1801 Research Blvd Ste 605, Rockville, MD 20850, USA; tzu-chun.lin@nih.gov

**Keywords:** breastfeeding, nutrition, early infant feeding practices, overweight, childhood obesity, complementary feeding, child development

## Abstract

Early infant growth trajectories have been linked to obesity risk. The aim of this study was to examine early infant feeding practices in association with anthropometric measures and risk of overweight/obesity in childhood. A total of 2492 children from Upstate KIDS, a population-based longitudinal cohort, were included for the analysis. Parents reported breastfeeding and complementary food introduction from 4 to 12 months on questionnaires. Weight and height were reported at 2–3 years of age and during later follow-up at 7–9 years of age. Age and sex z-scores were calculated. Linear mixed models were conducted, adjusting for maternal and child sociodemographic factors. Approximately 54% of infants were formula-fed at <5 months of age. Compared to those formula-fed, BMI- (adjusted B, −0.23; 95% CI: −0.42, −0.05) and weight-for-age z-scores (adjusted B, −0.16; −0.28, −0.03) were lower for those exclusively breastfed. Infants breastfed for ≥12 months had a lower risk of being overweight (aRR, 0.33; 0.18, 0.59) at 2–3 years, relative to formula-fed infants. Compared to introduction at <5 months, the introduction of fruits and vegetables between 5 and 8 months was associated with lower risk of obesity at 7–9 years (aRR, 0.45; 0.22, 0.93). The type and duration of breastfeeding and delayed introduction of certain complementary foods was associated with lower childhood BMI.

## 1. Introduction

The prevalence of childhood obesity continues to rise and approximately one in five children in the U.S. were obese between 2017 and 2020 [1]. Early infant growth has been linked to long-term health, as rapid early weight gain has been associated with a greater risk of coronary heart disease and diabetes [2,3,4]. One of the most important modifiable factors associated with early growth and later an overweight status and obesity is the type of infant feeding [5]. Evidence suggests an inverse association of exclusive breastfeeding with body mass index (BMI) and risk of childhood overweight compared to formula-fed infants [6,7,8]. The American Academy of Pediatrics (AAP) and the World Health Organization (WHO) recommends exclusive breastfeeding for the first 6 months of life followed by continued breastfeeding beyond one year, even after complementary foods are introduced around 6 months [9,10,11]. In 2022, the AAP recommended extending breastfeeding until 2 years of age [12]. Despite the well-known benefits, only 1 in 4 women in the U.S. exclusively breastfeed for at least 6 months, owing to different barriers including lack of prenatal education, lactation challenges, lack of support for breastfeeding in the workplace for working mothers, and an early introduction of complementary foods [13,14].

The AAP and WHO define complementary foods as involving the consumption of solid and liquid foods other than breast milk or infant formula, including the introduction of non-human milk and fruit juice or sugar-sweetened beverages (SSBs) [9,11]. The introduction of complementary foods serves to meet the individualized nutritional needs of the infant [15], although the age at which complementary foods are introduced may impact the infant’s growth and dietary patterns in early childhood [16,17,18,19]. Emerging evidence suggests that an early introduction of complementary foods (prior to 6 months of age) is associated with greater risk of obesity during childhood, yet findings are inconsistent [8,20,21,22,23]. A systematic review of 23 studies found that most showed no clear associations between the timing of the introduction to complementary foods and risk of obesity [23]. A large systematic review evaluated optimal timing of complementary foods with various outcomes [24]. Among four randomized controlled trials (RCTs), they found no impact between timing of complementary food introduction and risk of an overweight status, although studies were limited by small sample sizes (<500 in total). On the other hand, their meta-analysis of four observational studies of >9000 children showed that an earlier introduction of complementary foods increased the odds of an overweight status and obesity (OR: 1.34; 95% CI: 1.09, 1.65). These conflicting results suggest the need for additional studies to address gaps in the existing evidence, including examining specific foods.

In summary, evidence remains mixed on whether early complementary feeding impacts risk of childhood obesity. We hypothesize that while longer breastfeeding will be protective, any early complementary feeding will be putative regardless of the type of complementary food introduced. Most previous studies did not have prospective information on early infant feeding practices and many of the studies examining early infant feeding practices did so across different countries [7,23]. In addition, most studies have grouped each type of complementary food together to represent the timing of solid foods, making the assumption that fruits and vegetables are similar to grains and proteins in their impact on childhood obesity [24]. Additional prospective information is needed about feeding practices in the United States. Evaluating each of the early infant feeding practices and disaggregating the complementary foods is essential in understanding their implications for child weight in early and middle childhood. Therefore, the aim of this study is to examine early infant feeding practices and child anthropometric measures in children from the Upstate KIDS cohort at 2–3 and 7–9 years of age.

## 2. Methods

### 2.1. Study Population

Upstate KIDS is a prospective cohort study that enrolled 5034 mothers and 6171 infants born between 2008 and 2010 in New York State (excluding New York City) [25]. The study was designed to determine the effects of infertility treatment on childhood development and growth [25]. Singleton infants conceived by infertility treatment were identified based on birth certificate data and were frequency-matched (3:1) to infants without treatment by the perinatal region of birth [25]. For this analysis, a subset of singletons and twins from the main cohort with information on weight or height at 2–3 (*n* = 2492) and 7–9 years of age (*n* = 1633) were included. The New York State Department of Health and the University at Albany (State University of New York) Institutional Review Board (IRB) approved this study (NYSDOH IRB #07-097; UAlbany #08-179 and #15E-122) for first-phase approval (27 May 2008) and second-phase approval (1 May 2015) and served as the IRB designated by the National Institutes of Health under a reliance agreement. Parents provided written informed consent prior to enrollment.

### 2.2. Measurements

#### 2.2.1. Duration and Type of Breastfeeding

Parents completed questionnaires on their feeding practices at 4–5, 8, and 12 months. At each time point, parents reported whether they were breastfeeding and/or formula feeding. Distributions of the data were examined to determine the categories of the type and duration of breastfeeding. Exclusive breastfeeding was defined as a mother feeding only breastmilk at <5 months of age, partial breastfeeding was defined as a mother feeding a combination of breastmilk and formula, and formula feeding was defined as feeding only formula at <5 months of age. The duration of breastfeeding was determined by the dates mothers reported stopping breastfeeding and the last questionnaire on which they had indicated their child was continuously breastfeeding. The duration of breastfeeding was then categorized as <6, 6–<12, and ≥12 months in line with AAP and WHO guidelines [10,11].

#### 2.2.2. Introduction of Complementary Foods

The introduction of complementary foods was assessed on questionnaires at 4, 8, and 12 months. Parents reported which categories of solid foods and/or beverages were introduced: grains (cereal in a bottle, rice and other cereals, and biscuits); fruits or vegetables (pureed or solid table food); dairy (cow’s milk, goat milk, soy milk, unpasteurized milk, cheese, and other dairy foods); and protein (meats and eggs). The distribution of risk of obesity was examined across all categories as categorical variables (<5, 5–8, and 9–12 months) as some foods have been introduced at <5 months of age or were introduced between 5 and 8 and 9 and 12 months of age (e.g., grains, fruits and vegetables, and dairy). Timing of protein was dichotomized as infants being introduced between 5 and 8 months of age and between 9 and 12 months of age as our study did not ask about the introduction of protein separately on the 4-month questionnaire.

#### 2.2.3. Anthropometrics

Mothers were asked to report their child’s height and weight, as measured at pediatrician well-child office visits, between 24 and 36 months on questionnaires issued at 24, 30, and 36 months of age [26]. To aid recall, at the beginning of this study, parents were given a journal in which to write these values when they attended pediatric visits. Anthropometric measures were reported for 2492 children overall, and 1180 (47.4%) at one, 1006 (40.4%) at two, 281 (11.3%) at three, 23 (0.9%) at four, and 2 (0.1%) at five pediatric visits. During follow-up at 7, 8, and 9 years of age, mothers continued to report child’s height and weight at their child’s pediatric visits [26,27]. A total of 1633 children had measurements overall, and 843 (51.6%) at one, 506 (31.0%) at two, and 284 (17.4%) at three time points. BMI was calculated as kg/m^2^. From these measurements, age- and sex-standardized weight-, height-, and BMI-for-age z-scores were calculated using the WHO reference [28,29].

#### 2.2.4. Covariates

Information on covariates was obtained primarily from birth certificates and maternal questionnaires. Birth certificates provided the maternal and paternal age, private health insurance status, participation in the Special Supplemental Nutrition Program for Women, Infants, and Children (WIC), maternal pre-pregnancy height and weight, parity, plurality, and gestational age. At the time of enrollment, maternal race/ethnicity (non-Hispanic White, non-Hispanic Black, Asian, Hispanic, other), education (less than high school, high school or GED, some college, college, advanced degree), and smoking during pregnancy were obtained during the baseline questionnaire by 4 months postpartum. As we previously observed, a range of child and maternal factors has been associated with child growth and obesity; hence, the above factors were considered in the current analyses [15,19,20].

#### 2.2.5. Statistical Analyses

Descriptive statistics of maternal and child characteristics were calculated to evaluate distributions of the demographic characteristics of participants by the type of infant feeding. The associations of early feeding practices and repeatedly assessed anthropometric measures were estimated using linear mixed models. To estimate mean differences and 95% CIs for BMI-, weight-, and height-for-age z-scores, outcomes were modelled continuously. Risks of dichotomous outcomes of developing an overweight status (85th to <95th percentile) and obesity (≥95th percentile) were assessed using Poisson regression models to estimate risk ratios and their 95% CIs at 2–3 and 7–9 years of age, based on the International Obesity Task Force cut points [30]. The normal weight group (<85th percentile) served as the reference group in those models (hence, risk of an overweight status excluded individuals who were obese). For the overweight-risk analysis, a subset of singletons and twins (one randomly selected) at 2–3 (*n* = 1620) and 7–9 (*n* = 1194) years of age were included in the model due to lack of convergence when both twins were included. All other models included a random effect to account for the dependence between twins in the same family and were adjusted for the mother’s age, race/ethnicity, education, insurance status, smoking, pre-pregnancy BMI, child’s gestational age, plurality, and Women, Infants, and Children (WIC) participation. Because infants conceived by fertility treatment and twins were oversampled in this study, we used sampling weights in each model to account for this study design feature [25]. To account for missing exposure and covariate data, 20 data sets were created using multiple imputations by the chained equations method [31]. Sampling and inverse probability of treatment weights were multiplied for analyses to account for study design and for nonresponses to follow-up at 2–3 and 7–9 years, respectively.

Sensitivity analyses were performed. First, we ran linear mixed models to further examine mean differences for BMI-for-age z-scores at 7–9 years of age stratified by plurality, as previous literature suggests that twins have lower weight and height [32]. Second, we re-ran all models, additionally adjusting for timing of juice introduction, as we previously observed that earlier introduction to juice consumption increased the risk of childhood obesity in this cohort [26,33]. Analyses were conducted using SAS 9.4 (SAS Institute Inc., Cary, NC, USA).

## 3. Results

Approximately 20% of infants were exclusively breastfed, 26% partially breastfed, and 54% formula-fed at <5 months of age. Mothers who formula-fed were younger, more likely to smoke during pregnancy, less likely to be married or living as married, and more likely to have gestational hypertension and diabetes, and had higher twinning and pre-pregnancy BMI. When examining socioeconomic indicators, mothers that formula-fed had lower educational attainment, were less likely to have private health insurance, and had higher WIC participation (Table 1). At 2–3 years of age, 13.1% and 5.8% were at risk of developing an overweight status and obesity, respectively. At 7–9 years of age, 13.7% and 8.5% were at risk of developing an overweight status and obesity, respectively (Supplemental Table S1).

### 3.1. Type of Infant Feeding

**Figure 1:**
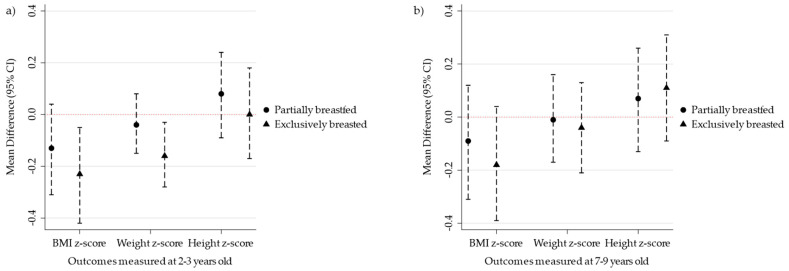
Type of infant feeding and anthropometric indicators at 2–3 (*n* = 2492) and 7–9 (*n* = 1633) years of age compared to formula-fed infants (reference), Upstate KIDS cohort *. * Type of feeding and anthropometric indicators at 2–3 (**a**) and 7–9 (**b**) years of age. The figure presents a scatter plot of the adjusted mean differences (95% confidence intervals) shown in Table 2 and Table 3 examining the differences in partially and exclusively breastfed groups when compared to the reference group of infants that were formula-fed only. The circle symbols represent infants partially breastfed; the triangle symbols represent infants exclusively breastfed.

At 2–3 years of age, compared to those formula feeding, children exclusively breastfeeding at <5 months of age had lower BMI-for-age z-scores (adjusted B, −0.23; −0.42, −0.05) and weight-for-age z-scores (adjusted, −0.16; −0.28, −0.03), but not height-for-age z-scores (adjusted B, 0.0004; −0.17, 0.18). Partial breastfeeding followed a similar trend (adjusted B, −0.13 BMI z-score; 95% CI: −0.31, 0.04) albeit attenuated after adjustment for sociodemographic factors (Table 2). The adjusted model is presented to show mean differences in the type of feeding and anthropometric indicators at 2–3 (Figure 1a) and 7–9 (Figure 1b) years of age. At 7–9 years of age, protective associations for exclusive and partial breastfeeding were attenuated for all outcomes after adjustment for covariates (Table 3).

Results at 2–3 years of age were congruent with findings of lower overweight risk for exclusive breastfeeding (RR, 0.51; 95% CI: 0.32, 0.80) compared to those formula-fed. Partial breastfeeding was also associated with a lower risk of an overweight status (RR, 0.62; 95% CI: 0.39, 0.96) (Table 4). While risks of obesity trended in the same direction, the estimates were less precise due to fewer numbers of children having obesity (Table 5).

### 3.2. Duration of Breastfeeding

**Figure 2:**
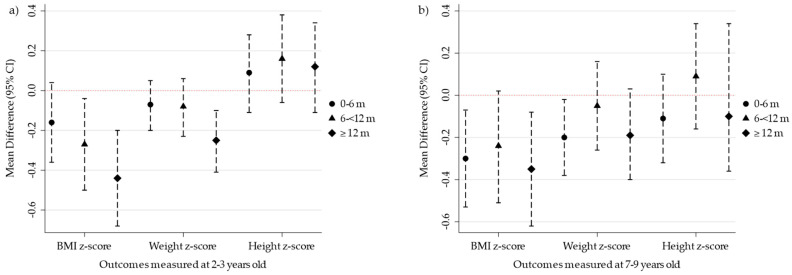
Duration of breastfeeding and anthropometric indicators at 2–3 (*n* = 2492) and 7–9 (*n* = 1633) years of age compared to formula-fed infants (reference), Upstate KIDS cohort *. * Duration of breastfeeding and anthropometric indicators at 2–3 (**a**) and 7–9 (**b**) years of age. The figure presents a scatter plot of the adjusted mean differences (95% confidence intervals) shown in Table 2 and Table 3 examining the differences in infants breastfed for 0–6 months, 6–<12 months, and ≥12 months when compared to the reference group of infants that were formula-fed only. The circle symbols represent infants breastfed for 0–6 months; the triangle symbols represent infants breastfed for 6–<12 months; and the diamond symbols represent infants breastfed for ≥12 months.

Children breastfed for ≥12 months (regardless of partial or exclusive) had lower BMI-for-age z-scores (adjusted B, −0.44; 95% CI: −0.68, −0.20) and weight-for-age z-scores (adjusted B, −0.25; 95% CI: −0.41, −0.10) compared to those formula-fed (Table 2). Breastfeeding for 12 or more months was also associated with lower risk of developing an overweight status (adjusted RR [aRR], 0.33; 95% CI: 0.18, 0.59) at 2–3 years of age. Breastfeeding for 6–<12 months was associated with lower BMI-for-age z-scores (adjusted B, −0.27; 95% CI: −0.50, −0.04) along with lower risk of developing an overweight status (aRR, 0.55; 95% CI: 0.32, 0.92) relative to those formula-fed. Associations for BMI-for-age z-scores remained at 7–9 years of age for those breastfeeding for ≥12 months (adjusted B, −0.35; 95% CI: −0.62, −0.08). At 7–9, children with an even low duration (<6 months) of any breastfeeding had lower BMI-for-age z-scores (adjusted B, −0.30; 95% CI: −0.53, −0.07) compared to formula feeding (Table 3). Figure 2 shows these adjusted mean differences in anthropometric indicators at 2–3 (Figure 2a) and 7–9 (Figure 2b) years of age.

### 3.3. Complementary Feeding

The distributions of the timing and type of complementary foods are provided in Supplemental Table S2. Approximately 59.7% of infants were introduced to grains at <5 months, while 63.8% and 48.0% were introduced to fruits and vegetables and protein between 5 and 8 months, respectively. Few infants had delayed introduction to these foods past 9 months of age, although 63.3% of infants were introduced to dairy during the latter part of infancy.

After adjusting for covariates, the timing of introducing any complementary food was not associated with anthropometric measures or overweight and obesity risk at 2–3 years (Table 2). Compared to those being introduced at <5 months, delaying introduction to between 5 and 8 months of age but not between 9 and 12 months was associated with lower BMI at 7–9 years of age (Table 3). However, this association was not corroborated with decreased overweight and obesity risk.

Although the timing of almost all specific complementary foods (i.e., fruits and vegetables, grains, and protein) was not associated with anthropometry at 2–3 years, the introduction of dairy was the exception. We observed higher BMI-for-age z-scores for those introduced to dairy between 5 and 8 months (adjusted B, 0.38; 95% CI: 0.10, 0.66) and between 9 and 12 months (adjusted B, 0.35; 95% CI: 0.09, 0.61) of age compared to those introduced at <5 months. Risk of developing an overweight status at 2–3 years of age was higher for those being introduced to dairy between 5 and 8 months (aRR, 2.84; 95% CI: 1.13, 7.13) and by 12 months (aRR, 3.42; 95% CI: 1.42, 8.27) of age (Table 4). Nevertheless, differences could be driven by the distribution of the timing of dairy introduction as a majority were introduced later rather than at <5 months of age (9.7%) or between 5 and 8 (26.8%) months of age like the other foods (Supplemental Table S2).

Associations in later childhood, however, reflect a different pattern. At 7–9 years of age, no persistent associations were found for BMI based on timing of dairy introduction, although significant associations remained for weight and introducing dairy between 5 and 8 months (adjusted B, 0.31; 95% CI: 0.07, 0.56) and 9 and 12 months (adjusted B, 0.26; 95% CI: 0.03, 0.48) of age compared to <5 months (Table 3). Conversely, lower BMI for delaying the introduction to fruits and vegetables (adjusted B, −0.37; 95% CI: −0.56, −0.18) as well as that for grains (adjusted B, −0.31; 95% CI: −0.49, −0.12) to between 5 and 8 months compared to <5 months became apparent. Children introduced to grains between 5 and 8 months also showed higher height-for-age z-scores (adjusted B, 0.19; 95% CI: 0.02, 0.36) relative to those with earlier introduction. The introduction of fruits and vegetables (aRR, 0.45; 95% CI: 0.22, 0.93) between 5 and 8 months also showed lower risks of developing obesity in middle childhood compared to earlier (Table 5).

### 3.4. Sensitivity Analysis

In a sensitivity analysis stratifying by the plurality status, results in both singletons and twins were consistent with our overall findings (Supplemental Table S3). In sensitivity analyses additionally adjusting for timing of juice introduction, results differed marginally (±0.02 units) and made no material difference in our inference for 2–3 (Supplemental Table S4) and 7–9 years of age (Supplemental Table S5).

## 4. Discussion

In our population-based, longitudinal cohort, we observed that breastfeeding was associated with lower BMI in both early and middle childhood, with a dose–response effect observed for an exclusive and longer duration of breastfeeding compared to formula feeding. Furthermore, we found a later introduction of select complementary foods between 5 and 8 months of age (such as fruits and vegetables and grains) to be associated with lower BMI whereas a later introduction of dairy was associated with higher BMI. Risk of an overweight status and obesity followed these BMI patterns, and the differences were driven by lower weight and no material differences in height.

Our findings regarding the type and duration of breastfeeding are consistent with previous studies, including a recent meta-analysis of 26 studies by Qiao et al. that evaluates breastfeeding and risk of obesity [7]. They found that those exclusively breastfed had a 47% lower risk of early childhood obesity compared to those formula-fed based on four studies [7]. They also found that those breastfeeding for more than 6 months had a 33% reduction in the risk of obesity compared to those breastfed for shorter durations based on five studies. We observed similar magnitudes of associations in reduction in risk for both early and middle childhood obesity. Furthermore, another meta-analysis by Yan et al. reported similar associations between those breastfed for more than 7 months and a lower risk of obesity [34].

Several studies were also consistent with our findings between breastfeeding at <5 months and BMI in early and middle childhood. Mantzorou et al. found that children (*n* = 2515) exclusively breastfed for at least 4 months had a lower BMI and a lower likelihood of being overweight or obese at 2–5 years of age (HR: 2.07; 95% CI: 1.70–2.40) [35]. In a prospective study including 13,401 children, Morgen et al. found not exclusively breastfeeding for at least 4 months or more to be associated with a higher BMI and higher odds of being overweight (OR: 3.67; 95% CI: 2.10, 6.43) at 7 years of age [36].

There are several biological pathways explaining the relationship between breastfeeding and childhood obesity. One pathway may involve the bioactive factors contained in breastmilk, such as lactoferrin, oligosaccharides, long-chained polyunsaturated fatty acids, glycoproteins, and secretory IgA antibodies that may control nutrient use and play a vital role in regulating metabolism [37]. In addition, breastmilk contains other biologically active factors, namely hormones, such as adiponectin, leptin, ghrelin, and insulin-like growth factor-1. One hormone commonly examined is leptin, an adipocyte-derived hormone known for controlling the regulation of food intake and energy balance, which has been associated with weight gain during infancy and early-life programming of obesity [37,38]. In a cross-sectional study conducted by Savino et al. in 36 exclusively breastfed infants, infant serum leptin concentrations were positively correlated with infant weight [39]. Another study conducted by Miralles et al. found similar results in a group of 28 women breastfeeding their infants for at least 6 months that showed a strong negative correlation between milk leptin concentrations at 1 and 3 months of lactation and infant BMI from 12 to 24 months of age [40]. This dose–response effect of leptin concentrations and obesity development can also be further described in rodent models [41]. Overall, this may suggest that bioactive factors found in breastmilk may have a protective effect against later risk of an overweight status and obesity.

Studies evaluating timing of the introduction of complementary foods and risk of childhood overweight and obese statuses have had mixed results. A systematic review by Pearce et al. included 23 studies evaluating the timing of complementary foods and risk of obesity [23]. They found that most of the previous studies showed no clear association between the timing of complementary foods and obesity. Similar to our findings, most studies presented significant associations prior to the adjustment of important confounders, which may highlight the influence of sociodemographic and maternal characteristics on early infant feeding patterns and later development of overweight and obesity. In our study, we did find an association between delaying fruits and vegetables and grains to between 5 and 8 months of age compared to <5 months and lower BMI in middle childhood. This is consistent with four studies reported by Pearce et al. that showed delaying the introduction of complementary foods was associated with lower BMI after adjustment [22,42,43,44]. However, most recent studies failed to examine the timing of different complementary foods as most examine timing of overall complementary foods. Another systematic review conducted by Pearce et al. identified 10 studies evaluating the timing of different complementary foods and BMI in children in early and late childhood. They found that none of the studies observed an effect between the different types of foods introduced and subsequent BMI in childhood except for one prospective study finding a similar association between a later introduction of grains around 8 months of age and lower BMI at 12 months of age [45,46]. Lastly, several studies defined late introduction as early as 4 months of age, not in keeping with the AAP recommendations [11], which makes it difficult to interpret their overall findings [23,47,48,49].

The AAP recommends delaying the introduction of dairy and other dairy products until 12 months of age and our study showed that approximately 27.3% of children were already introduced between 5 and 8 months [11]. Meanwhile, our study did not find those with delayed introduction to dairy between 9 and 12 months of age to have lower BMI and risk of developing an overweight status in early childhood when compared to earlier introduction. It is unclear why an earlier introduction might have a protective effect as approximately 9.4% reported dairy consumption at <5 months of age. However, we did not see associations persist in middle childhood. In the context of existing literature, studies have found an association between high dairy consumption and increased BMI and obesity risk during the latter part of infancy [50,51]. In a prospective cohort, Gunther et al. found in 203 children that high dairy consumption at 12 months of age was associated with higher BMI (mean difference, 0.43; 95% CI: 0.17, 0.70) and risk of an overweight status (OR: 2.74; 95% CI: 1.36, 5.52) at 7 years of age [51]. This association may be explained by the notable difference in the nutritional composition of breastmilk and dairy products. A meta-analysis conducted by Arnesen et al. revealed that higher protein intake from dairy products during infancy was associated with significantly higher BMI and risk of an overweight status and obesity compared to those only breastfed [52]. Taken together, these findings may suggest that timing of dairy plays an intricate role in both children’s dietary and growth patterns. However, future research is needed to understand how an earlier introduction of dairy influences patterns of dairy consumption during middle childhood.

The strengths of this study include the longitudinal design and follow-up throughout early and middle childhood, although our study has several limitations. First, measurement error may be introduced by maternal reporting of the child’s height and weight on questionnaires. This potential limitation is minimized by asking mothers to report the child’s height and weight recorded at their child’s wellness visits. Second, the questionnaire was unable to distinguish the frequency or quantity of the solid foods and beverages consumed when assessing timing of complementary foods. Though this limitation is similarly noted in previous studies [23], this study was strengthened by its ability to assess the infant’s intake of types of complementary foods consumed longitudinally versus studies that ask mothers to recall the age of introduction of complementary foods retrospectively at a single time point. This does suggest, however, that further studies are needed to continue to assess early infant feeding practices and their relationships to childhood BMI to ascertain how timing of certain foods or nutrients impacts growth and future dietary preferences. Third, due to the nonresponse to follow-up, this study may have some selection bias; however, we used inverse probability weights to mitigate this. Last, our cohort is atypical of the general U.S. population as our cohort was predominately non-Hispanic White and of a higher socioeconomic status, which may limit the generalizability of the study findings.

## 5. Conclusions

In our longitudinal birth cohort, we observed associations of the type and duration of breastfeeding and the delay of different complementary foods with anthropometric measures in early and middle childhood. Our findings are also consistent with previous studies examining the influence of sociodemographic and maternal factors on early infant feeding practices and anthropometric measures in childhood. Future studies are needed to continue to evaluate the timing of different types of complementary foods introduced and growth along with examining the establishment of long-term dietary patterns in later childhood.

## Figures and Tables

**Table 1:** Maternal and infant characteristics; entire cohort (*n* = 2492) and by infant feeding at <5 months in the Upstate KIDS cohort *.

		Type of Infant Feeding			
Overall, *n*, %	Entire Cohort	EBF	PF	FF	
	(*n* = 2492)	(*n* = 496, 20%)	(*n* = 644, 26%)	(*n* = 1352, 54%)	*p*-Value ^†^
**Maternal characteristics**					
Age, y, mean ± SD	31.9 ± 5.6	32.0 ± 5.3 ^a^	32.5 ± 5.2 ^a^	31.6 ± 5.8 ^b^	0.003
Race/ethnicity					<0.0001
Non-Hispanic White	2153 (86.4)	432 (87.1)	553 (85.9)	1168 (86.4)	
Non-Hispanic Black	51 (2.1)	4 (0.8)	18 (2.8)	29 (2.1)	
Asian	86 (3.5)	29 (5.9)	31 (4.8)	26 (1.9)	
Hispanic	143 (5.7)	19 (3.8)	34 (5.3)	90 (6.7)	
Other	59 (2.4)	12 (2.4)	8 (1.2)	39 (2.9)	
Education					
Less than high school	48 (1.9)	4 (0.8)	6 (0.9)	38 (2.8)	<0.0001
High school or GED equivalent	171 (6.9)	24 (4.8)	30 (4.7)	117 (8.7)	
Some college	605 (24.3)	85 (17.1)	122 (18.9)	398 (29.4)	
College	668 (26.8)	142 (28.6)	210 (32.6)	316 (23.4)	
Advanced degree	1000 (40.1)	241 (48.6)	276 (42.9)	483 (35.7)	
Private insurance	2145 (86.1)	434 (87.5)	580 (90.1)	1131 (83.7)	0.0003
WIC recipient	375 (15.2)	47 (9.6)	77 (12.2)	251 (18.7)	<0.0001
Married or living as married	2327 (93.4)	482 (97.2)	604 (93.8)	1241 (91.8)	0.0002
Smoking during pregnancy	170 (6.8)	17 (3.4)	25 (3.9)	128 (9.5)	<0.0001
Alcohol during pregnancy	340 (13.6)	83 (16.7)	115 (17.9)	142 (10.5)	<0.0001
Height, cm, mean ± SD	164.5 ± 7.1	164.7 ± 7.0	164.8 ± 7.1	164.3 ± 7.2	0.330
Weight, kg, mean ± SD	73.8 ± 17.9	69.1 ± 15.5 ^a^	71.6 ± 15.9 ^a^	76.6 ± 19.1 ^b^	<0.0001
Pre-pregnancy BMI, kg/m^2^, mean ± SD	26.6 ± 6.5	24.8 ± 5.1 ^a^	25.8 ± 5.6 ^b^	27.6 ± 7.1 ^c^	<0.0001
Gestational diabetes	240 (9.6)	36 (7.3)	53 (8.2)	151 (11.2)	0.016
Gestational hypertension	294 (11.8)	33 (6.7)	69 (10.7)	192 (14.2)	<0.0001
Fertility treatment use	982 (39.4)	169 (34.1)	278 (43.2)	535 (39.6)	0.008
**Infant characteristics**					
Child sex, male	1251 (50.2)	253 (51.0)	327 (50.8)	671 (49.6)	0.823
Birth weight, g, mean ± SD	3132 ± 678	3373 ± 549 ^a^	3167 ± 684 ^b^	3026 ± 694 ^c^	<0.0001
Plurality, twin	798 (32.0)	62 (12.5)	184 (28.6)	552 (40.8)	<0.0001
Gestational age, mean ± SD	38.0 ± 2.3	38.7 ± 1.7 ^a^	38.1 ± 2.4 ^b^	37.6 ± 2.4 ^c^	<0.0001

Abbreviations: GED, general educational diploma; EBF, exclusively breastfeeding; PF, partially breastfeeding; FF, formula feeding; WIC, Women, Infants, and Children. * Values are *n* (%) unless otherwise noted. ^†^ *p*-values were calculated using the χ^2^ test for categorical variables and one-way ANOVA test for continuous variables. ^a–c^ Different superscript letters mean that pairwise comparisons were significantly different across rows at the α = 0.05 level in Tukey post hoc tests.

**Table 2:** Childhood anthropometric indicators at 2–3 years of age (*n* = 2492) based on early feeding practices, Upstate KIDS cohort.

	BMI-for-Age z-Scores		Weight-for-Age z-Scores		Height-for-Age z-Scores	
	Unadjusted *	Adjusted ^†^	Unadjusted *	Adjusted ^†^	Unadjusted *	Adjusted ^†^
Exposure	Mean Difference (95% CI)	Mean Difference (95% CI)	Mean Difference (95% CI)	Mean Difference (95% CI)	Mean Difference (95% CI)	Mean Difference (95% CI)
**Type of infant feeding**						
Formula feeding	Ref	Ref	Ref	Ref	Ref	Ref
Partial breastfeeding	−0.23 (−0.41, −0.06)	−0.13 (−0.31, 0.04)	−0.08 (−0.19, 0.03)	−0.04 (−0.15, 0.08)	0.12 (−0.04, 0.29)	0.08 (−0.09, 0.24)
Exclusive breastfeeding	−0.31 (−0.49, −0.13)	−0.23 (−0.42, −0.05)	−0.16 (−0.28, −0.05)	−0.16 (−0.28, −0.03)	0.08 (−0.09, 0.24)	0.0004 (−0.17, 0.18)
**Duration of breastfeeding**						
None (formula feeding)	Ref	Ref	Ref	Ref	Ref	Ref
<6 months	−0.19 (−0.40, 0.01)	−0.16 (−0.36, 0.04)	−0.08 (−0.21, 0.05)	−0.07 (−0.20, 0.05)	0.12 (−0.07, 0.31)	0.09 (−0.11, 0.28)
6–<12 months	−0.36 (−0.59, −0.14)	−0.27 (−0.50, −0.04)	−0.12 (−0.26, 0.03)	−0.08 (−0.23, 0.06)	0.22 (0.01, 0.43)	0.16 (−0.06, 0.38)
≥12 months	−0.56 (−0.79, −0.33)	−0.44 (−0.68, −0.20)	−0.29 (−0.44, −0.14)	−0.25 (−0.41, −0.10)	0.19 (−0.03, 0.41)	0.12 (−0.11, 0.34)
**Introduction of complementary foods**						
<5 months	Ref	Ref	Ref	Ref	Ref	Ref
5–8 months	−0.16 (−0.31, −0.01)	−0.04 (−0.19, 0.11)	−0.14 (−0.24, −0.05)	−0.08 (−0.18, 0.01)	−0.09 (−0.23, 0.05)	−0.11 (−0.25, 0.03)
9–12 months	0.03 (−0.58, 0.64)	0.18 (−0.42, 0.78)	−0.06 (−0.45, 0.32)	−0.01 (−0.39, 0.36)	−0.09 (−0.69, 0.51)	−0.17 (−0.78, 0.43)
**Dairy**						
<5 months	Ref	Ref	Ref	Ref	Ref	Ref
5–8 months	0.27 (−0.02, 0.55)	0.38 (0.10, 0.66)	0.12 (−0.06, 0.30)	0.16 (−0.02, 0.34)	−0.09 (−0.36, 0.17)	−0.13 (−0.39, 0.14)
9–12 months	0.29 (0.02, 0.55)	0.35 (0.09, 0.61)	0.04 (−0.13, 0.20)	0.05 (−0.11, 0.21)	−0.19 (−0.44, 0.05)	−0.21 (−0.46, 0.03)
**Fruits and vegetables**						
<5 months	Ref	Ref	Ref	Ref	Ref	Ref
5–8 months	−0.25 (−0.41, −0.09)	−0.12 (−0.28, 0.04)	−0.15 (−0.26, −0.05)	−0.07 (−0.18, 0.03)	−0.02 (−0.17, 0.13)	−0.04 (−0.19, 0.11)
9–12 months	−0.07 (−0.48, 0.34)	−0.04 (−0.44, 0.36)	−0.08 (−0.33, 0.17)	−0.05 (−0.29, 0.20)	−0.06 (−0.47, 0.35)	−0.05 (−0.45, 0.35)
**Grains**						
<5 months	Ref	Ref	Ref	Ref	Ref	Ref
5–8 months	−0.17 (−0.32, −0.02)	−0.03 (−0.19, 0.12)	−0.14 (−0.24, −0.04)	−0.08 (−0.18, 0.02)	−0.05 (−0.19, 0.09)	−0.09 (−0.23, 0.06)
9–12 months	0.08 (−0.33, 0.49)	0.17 (−0.22, 0.57)	−0.07 (−0.32, 0.19)	0.02 (−0.23, 0.27)	−0.17 (−0.57, 0.23)	−0.11 (−0.51, 0.29)
**Protein**						
5–8 months	Ref	Ref	Ref	Ref	Ref	Ref
9–12 months	0.07 (−0.09, 0.22)	0.10 (−0.05, 0.25)	−0.06 (−0.16, 0.03)	−0.04 (−0.13, 0.06)	−0.12 (−0.26, 0.02)	−0.11 (−0.24, 0.03)

Ref = Reference group. * Values are mean differences and their 95% confidence intervals from a linear mixed model. ^†^ Adjusted for mother’s age, race/ethnicity, education, insurance status, smoking, pre-pregnancy BMI, child’s gestational age, multiple birth status and Women, Infants, and Children (WIC) participation.

**Table 3:** Childhood anthropometric indicators at 7–9 years of age (*n* = 1633) based on early feeding practices, Upstate KIDS cohort.

	BMI-for-Age z-Score		Weight-for-Age z-Score		Height-for-Age z-Score	
	Unadjusted *	Adjusted ^†^	Unadjusted *	Adjusted ^†^	Unadjusted *	Adjusted ^†^
Exposure	Mean Difference (95% CI)	Mean Difference (95% CI)	Mean Difference (95% CI)	Mean Difference (95% CI)	Mean Difference (95% CI)	Mean Difference (95% CI)
**Type of infant feeding**						
Formula feeding	Ref	Ref	Ref	Ref	Ref	Ref
Partial breastfeeding	−0.34 (−0.55, −0.13)	−0.09 (−0.31, 0.12)	−0.22 (−0.39, −0.05)	−0.01 (−0.17, 0.16)	0.02 (−0.17, 0.21)	0.07 (−0.13, 0.26)
Exclusive breastfeeding	−0.44 (−0.65, −0.23)	−0.18 (−0.39, 0.04)	−0.21 (−0.38, −0.05)	−0.04 (−0.21, 0.13)	0.12 (−0.06, 0.31)	0.11 (−0.09, 0.31)
**Duration of breastfeeding**						
None (formula feeding)	Ref	Ref	Ref	Ref	Ref	Ref
<6 months	−0.45 (−0.69, −0.22)	−0.30 (−0.53, −0.07)	−0.32 (−0.5, −0.13)	−0.20 (−0.38, −0.02)	−0.12 (−0.33, 0.08)	−0.11 (−0.32, 0.10)
6–<12 months	−0.57 (−0.83, −0.31)	−0.24 (−0.51, 0.02)	−0.31 (−0.52, −0.10)	−0.05 (−0.26, 0.16)	0.08 (−0.16, 0.31)	0.09 (−0.16, 0.34)
≥12 months	−0.71 (−0.98, −0.44)	−0.35 (−0.62, −0.08)	−0.45 (−0.66, −0.23)	−0.19 (−0.40, 0.03)	−0.12 (−0.36, 0.12)	−0.10 (−0.36, 0.15)
**Introduction of complementary foods**						
<5 months	Ref	Ref	Ref	Ref	Ref	Ref
5–8 months	−0.48 (−0.65, −0.30)	−0.27 (−0.45, −0.10)	−0.28 (−0.42, −0.13)	−0.13 (−0.26, 0.01)	0.10 (−0.06, 0.26)	0.12 (−0.05, 0.28)
9–12 months	−0.16 (−0.86, 0.54)	0.19 (−0.47, 0.85)	−0.12 (−0.69, 0.45)	0.11 (−0.42, 0.65)	−0.01 (−0.61, 0.59)	0.07 (−0.53, 0.67)
**Dairy**						
<5 months	Ref	Ref	Ref	Ref	Ref	Ref
5–8 months	0.14 (−0.20, 0.47)	0.26 (−0.06, 0.58)	0.19 (−0.07, 0.46)	0.31 (0.07, 0.56)	0.11 (−0.19, 0.41)	0.10 (−0.20, 0.39)
9–12 months	0.18 (−0.13, 0.49)	0.27 (−0.02, 0.56)	0.16 (−0.08, 0.40)	0.26 (0.03, 0.48)	0.07 (−0.21, 0.34)	0.06 (−0.21, 0.33)
**Fruits and vegetables**						
<5 months	Ref	Ref	Ref	Ref	Ref	Ref
5–8 months	−0.54 (−0.73, −0.34)	−0.37 (−0.56, −0.18)	−0.35 (−0.50, −0.20)	−0.24 (−0.39, −0.09)	0.02 (−0.16, 0.19)	0.01 (−0.16, 0.19)
9–12 months	−0.18 (−0.61, 0.26)	−0.12 (−0.54, 0.29)	−0.21 (−0.57, 0.15)	−0.18 (−0.52, 0.16)	−0.14 (−0.51, 0.24)	−0.15 (−0.53, 0.23)
**Grains**						
<5 months	Ref	Ref	Ref	Ref	Ref	Ref
5–8 months	−0.49 (−0.68, −0.31)	−0.31 (−0.49, −0.12)	−0.23 (−0.38, −0.09)	−0.10 (−0.25, 0.04)	0.17 (0.01, 0.33)	0.19 (0.02, 0.36)
9–12 months	−0.38 (−0.83, 0.08)	−0.08 (−0.51, 0.34)	−0.31 (−0.67, 0.06)	−0.10 (−0.44, 0.24)	−0.05 (−0.43, 0.34)	0.07 (−0.32, 0.46)
**Protein**						
5–8 months	Ref	Ref	Ref	Ref	Ref	Ref
9–12 months	−0.02 (−0.20, 0.17)	0.01 (−0.17, 0.19)	−0.02 (−0.16, 0.13)	−0.01 (−0.15, 0.12)	0.0005 (−0.16, 0.16)	0.004 (−0.17, 0.16)

Ref = Reference group. * Values are mean differences and their 95% confidence intervals from a linear mixed model. ^†^ Adjusted for mother’s age, race/ethnicity, education, insurance status, smoking, pre-pregnancy BMI, child’s gestational age, multiple birth status, and Women, Infants, and Children (WIC) participation.

**Table 4:** Childhood risk of an overweight status (85th to <95th percentile) at 2–3 (*n* = 1620) and 7–9 (*n* = 1194) years of age based on early feeding practices, Upstate KIDS cohort.

	2–3 Years Old		7–9 Years Old	
	Unadjusted *	Adjusted ^†^	Unadjusted *	Adjusted ^†^
Exposure	RR (95% CI)	RR (95% CI)	RR (95% CI)	RR (95% CI)
**Type of infant feeding**				
Formula feeding	Ref	Ref	Ref	Ref
Partial breastfeeding	0.49 (0.31, 0.76)	0.62 (0.39, 0.96)	0.74 (0.46, 1.19)	0.91 (0.55, 1.49)
Exclusive breastfeeding	0.44 (0.29, 0.69)	0.51 (0.32, 0.80)	0.74 (0.47, 1.15)	0.93 (0.57, 1.50)
**Duration of breastfeeding**				
None (formula feeding)	Ref	Ref	Ref	Ref
<6 months	0.59 (0.38, 0.92)	0.65 (0.42, 1.01)	0.63 (0.39, 1.03)	0.68 (0.41, 1.12)
6–<12 months	0.45 (0.27, 0.75)	0.55 (0.32, 0.92)	0.62 (0.36, 1.08)	0.75 (0.42, 1.35)
≥12 months	0.27 (0.15, 0.47)	0.33 (0.18, 0.59)	0.49 (0.28, 0.87)	0.63 (0.34, 1.16)
**Introduction of complementary foods**				
<5 months	Ref	Ref	Ref	Ref
5–8 months	0.70 (0.49, 1.01)	0.86 (0.60, 1.25)	0.75 (0.51, 1.11)	0.87 (0.58, 1.31)
9–12 months	0.12 (0.00, 3.90)	0.14 (0.00, 5.49)	1.31 (0.33, 5.13)	1.91 (0.50, 7.25)
**Dairy**				
<5 months	Ref	Ref	Ref	Ref
5–8 months	2.59 (1.02, 6.57)	2.84 (1.13, 7.13)	0.78 (0.39, 1.56)	0.77 (0.39, 1.54)
9–12 months	3.21 (1.30, 7.88)	3.42 (1.42, 8.27)	0.94 (0.50, 1.75)	0.92 (0.50, 1.72)
**Fruits and vegetables**				
<5 months	Ref	Ref	Ref	Ref
5–8 months	0.71 (0.49, 1.02)	0.82 (0.57, 1.19)	0.66 (0.44, 0.99)	0.74 (0.49, 1.12)
9–12 months	0.29 (0.07, 1.30)	0.33 (0.08, 1.40)	1.57 (0.75, 3.30)	1.71 (0.81, 3.62)
**Grains**				
<5 months	Ref	Ref	Ref	Ref
5–8 months	0.64 (0.44, 0.93)	0.82 (0.57, 1.20)	0.75 (0.50, 1.12)	0.88 (0.58, 1.33)
9–12 months	0.55 (0.17, 1.74)	0.64 (0.21, 1.98)	1.51 (0.65, 3.51)	1.84 (0.79, 4.29)
**Protein**				
5–8 months	Ref	Ref	Ref	Ref
9–12 months	1.25 (0.87, 1.81)	1.29 (0.90, 1.85)	1.05 (0.71, 1.54)	1.10 (0.74, 1.62)

Ref = Reference group. * Values are risk ratios and their 95% confidence intervals from Poisson regression models. ^†^ Adjusted for mother’s age, race/ethnicity, education, insurance status, smoking, pre-pregnancy BMI, child’s gestational age, multiple statuses, and Women, Infants, and Children (WIC) participation.

**Table 5:** Childhood risk of obesity (≥95th percentile) at 2–3 (*n* = 2492) and 7–9 (*n* = 1633) years of age based on early feeding practices, Upstate KIDS cohort.

	2–3 Years Old		7–9 Years Old	
	Unadjusted *	Adjusted ^†^	Unadjusted *	Adjusted ^†^
Exposure	RR (95% CI)	RR (95% CI)	RR (95% CI)	RR (95% CI)
**Type of infant feeding**				
Formula feeding	Ref	Ref	Ref	Ref
Partial breastfeeding	0.70 (0.33, 1.49)	0.85 (0.42, 1.73)	0.47 (0.19, 1.20)	0.69 (0.29, 1.67)
Exclusive breastfeeding	0.42 (0.18, 0.98)	0.60 (0.27, 1.33)	0.28 (0.10, 0.76)	0.51 (0.20, 1.31)
**Duration of breastfeeding**				
None (formula feeding)	Ref	Ref	Ref	Ref
<6 months	0.74 (0.34, 1.65)	0.78 (0.37, 1.62)	0.63 (0.26, 1.51)	0.74 (0.33, 1.66)
6-<12 months	0.35 (0.13, 0.94)	0.45 (0.18, 1.15)	0.32 (0.10, 0.99)	0.56 (0.19, 1.60)
≥12 months	0.36 (0.13, 0.97)	0.50 (0.20, 1.28)	0.25 (0.08, 0.80)	0.52 (0.17, 1.60)
**Introduction of complementary foods**				
<5 months	Ref	Ref	Ref	Ref
5–8 months	0.47 (0.23, 0.94)	0.56 (0.30, 1.08)	0.40 (0.17, 0.91)	0.51 (0.24, 1.11)
9–12 months	0.71 (0.05, 9.34)	0.54 (0.03, 9.83)	0.72 (0.04, 13.98)	0.94 (0.05, 17.30)
**Dairy**				
<5 months	Ref	Ref	Ref	Ref
5–8 months	1.17 (0.32, 4.30)	1.24 (0.39, 3.94)	2.09 (0.35, 12.37)	2.71 (0.56, 13.18)
9–12 months	1.27 (0.37, 4.33)	1.26 (0.42, 3.74)	2.94 (0.55, 15.65)	4.29 (0.98, 18.79)
**Fruits and vegetables**				
<5 months	Ref	Ref	Ref	Ref
5–8 months	0.42 (0.22, 0.79)	0.49 (0.27, 0.89)	0.38 (0.18, 0.80)	0.45 (0.22, 0.93)
9–12 months	0.79 (0.17, 3.74)	0.89 (0.21, 3.70)	0.54 (0.10, 2.83)	0.52 (0.11, 2.52)
**Grains**				
<5 months	Ref	Ref	Ref	Ref
5–8 months	0.47 (0.23, 0.93)	0.59 (0.31, 1.11)	0.38 (0.17, 0.88)	0.48 (0.22, 1.06)
9–12 months	0.48 (0.06, 3.66)	0.36 (0.04, 3.59)	0.37 (0.04, 3.69)	0.41 (0.03, 4.98)
**Protein**				
5–8 months	Ref	Ref	Ref	Ref
9–12 months	1.12 (0.59, 2.12)	1.16 (0.65, 2.07)	1.04 (0.49, 2.20)	1.30 (0.65, 2.61)

Ref = Reference group. * Values are risk ratios and their 95% confidence intervals from Poisson regression models. ^†^ Adjusted for mother’s age, race/ethnicity, education, insurance status, smoking, pre-pregnancy BMI, child’s gestational age, multiple statuses, and Women, Infants, and Children (WIC) participation.

## Data Availability

The data that support the findings of this study are available from the corresponding author upon approval of a data use agreement. The data are not publicly available due to privacy or ethical restrictions.

## Supplementary Materials

The following supporting information can be downloaded at: https://www.mdpi.com/article/10.3390/nu16050714/s1, Table S1. Prevalence of developing overweight and obesity by type of infant feeding, Upstate KIDS cohort. Table S2. Entire cohort (*n* = 2492) based on timing of complementary foods, Upstate KIDS cohort. Table S3. Childhood anthropometric indicators at 7–9 years of age (*n* = 1633) based on early feeding practices stratified by plurality, Upstate KIDS cohort. Table S4. Childhood anthropometric indicators at 2–3 years of age (*n* = 2492) based on early feeding practices adjusted by age of juice introduction, Upstate KIDS cohort. Table S5. Childhood anthropometric indicators at 7–9 years of age (*n* = 1633) based on early feeding practices adjusted by age of juice introduction, Upstate KIDS cohort.
