# Correction: Clayton et al. Early Infant Feeding Practices and Associations with Growth in Childhood. *Nutrients* 2024, *16*, 714

**DOI:** 10.3390/nu17132171

**Published:** 2025-06-30

**Authors:** Priscilla K. Clayton, Diane L. Putnick, Ian R. Trees, Akhgar Ghassabian, Jordan N. Tyris, Tzu-Chun Lin, Edwina H. Yeung

**Affiliations:** 1 Epidemiology Branch, Division of Population Health Research, *Eunice Kennedy Shriver* National Institute of Child Health and Human Development, National Institutes of Health, 6710B Rockledge Dr, Bethesda, MD 20817, USA; priscilla.clayton@nih.gov (P.K.C.); putnickd@mail.nih.gov (D.L.P.); ian.trees@nih.gov (I.R.T.); 2 Department of Pediatrics and Population Health, NYU Grossman School of Medicine, 550 First Avenue, New York, NY 10016, USA; akhgar.ghassabian@nyulangone.org; 3 Division of Hospital Medicine, Children’s National Hospital, 111 Michigan Avenue NW, Washington, DC 20010, USA; jbarger@childrensnational.org; 4 Glotech Inc., 1801 Research Blvd Ste 605, Rockville, MD 20850, USA; tzu-chun.lin@nih.gov

## Removed Citation and Additional Reference

In the original publication [1], we removed the citation of ref. [20] in the first sentence of Section 2.2.4. No citation is needed for this sentence.

The citation of [20,23] in the last second sentence in the first paragraph of Section 2.2.5 has been changed to [31] and should read:

31. White, I.R.; Royston, P.; Wood, A.M. Multiple imputation using chained equations: Issues and guidance for practice. *Stat. Med.* **2011**, *30*, 377–399. https://doi.org/10.1002/sim.4067.

With this change, the new reference [31] has been added to the references section. The order of the rest of the references has been adjusted accordingly.

## Supplementary Materials Updated

With this correction, the Supplementary Materials have been updated to ensure consistency throughout the work.

The authors state that the scientific conclusions are unaffected. This correction was approved by the Academic Editor. The original publication has also been updated.
