# Supplementary material for: Early Infant Feeding Practices and Associations with Growth in Childhood
Source: Nutrients. 2024 Feb 29;16(5):714. doi: 10.3390/nu16050714 (PMC10934149; doi:10.3390/nu16050714)
Supplement: Supplementary file 1 [file nutrients-16-00714-s001.zip › nutrients-2865075-supplementary.pdf]

Supplemental Table S1. Prevalence of developing overweight and obesity by type of infant feeding, Upstate KIDS Cohort

Outcomes	Type of infant feeding*					
	n	Overall	EBF	PF	FF	*p*-Value †
**2-3y**	2492					
Developing overweight		224 (13.1)	31 (8.2)	47 (9.8)	146 (17.1)	<.0001
Developing obesity		106 (5.8)	15 (3.8)	24 (4.8)	67 (7.3)	0.024
**7-9y**	1633					
Developing overweight		132 (13.7)	31 (14.2)	28 (11.2)	73 (14.6)	0.433
Developing obesity		90 (8.5)	9 (4.0)	17 (6.4)	64 (11.4)	0.001

Abbreviations: EBF, exclusively breastfed; PF, partially breastfed; FF, formula-fed.
*Values are n (%) unless otherwise noted.
† *p*-values were calculated using the χ^2 test for categorical variables.

Supplemental Table S2. Entire cohort (n=2492) based on timing of complementary foods, Upstate KIDS cohort.

**Overall, n, %**	Timing of complementary foods		
	**<5 months**	**5-8 months**	**9-12 months**
Dairy	235 (9.4)	680 (27.3)	1577 (63.3)
Fruits & Vegetables	806 (32.3)	1589 (63.8)	97 (3.9)
Grains	1488 (59.7)	910 (36.5)	94 (3.8)
Protein	***	1197 (48.0)	1295 (52.0)

***Note: Separate questions about protein were not asked at <5 months.

Supplemental Table S3. Childhood anthropometric indicators at 7-9 years of age (n=1633) based on early feeding practices stratified by plurality, Upstate KIDS cohort

	Singletons (n=1150)		Twins (n=483)	
**Exposure**	Unadjusted* Mean difference (95% CI)	Adjusted† Mean difference (95% CI)	Unadjusted* Mean difference (95% CI)	Adjusted† Mean difference (95% CI)
**Type of infant feeding**				
Formula feeding	Ref	Ref	Ref	Ref
Partial breastfeeding	-0.40 (-0.65, -0.14)	-0.16 (-0.42, 0.10)	-0.33 (-0.64, -0.03)	0.03 (-0.25, 0.32)
Exclusive breastfeeding	-0.55 (-0.78, -0.31)	-0.21 (-0.45, 0.03)	-0.67 (-1.23, -0.12)	-0.38 (-0.89, 0.13)
**Duration of breastfeeding**				
None (formula feeding)	Ref	Ref	Ref	Ref
<6 months	-0.52 (-0.80, -0.24)	-0.37 (-0.65, -0.09)	-0.29 (-0.62, 0.03)	-0.11 (-0.41, 0.19)
6-<12 months	-0.67 (-0.98, -0.36)	-0.35 (-0.67, -0.04)	-0.53 (-0.94, -0.12)	0.02 (-0.37, 0.41)
≥12 months	-0.86 (-1.17, -0.56)	-0.46 (-0.78, -0.14)	-0.68 (-1.18, -0.18)	-0.23 (-0.69, 0.23)
**Introduction of complementary foods**				
<5 months	Ref	Ref	Ref	Ref
5-8 months	-0.44 (-0.65, -0.23)	-0.24 (-0.44, -0.03)	-0.54 (-0.83, -0.26)	-0.28 (-0.55, -0.01)
9-12 months	-0.27 (-1.04, 0.50)	0.17 (-0.57, 0.91)	0.40 (-1.11, 1.92)	0.32 (-0.96, 1.59)
**Dairy**				
<5 months	Ref	Ref	Ref	Ref
5-8 months	0.15 (-0.24, 0.54)	0.27 (-0.10, 0.65)	0.02 (-0.50, 0.55)	0.07 (-0.41, 0.55)
9-12 months	0.25 (-0.11, 0.61)	0.37 (0.02, 0.71)	0.07 (-0.40, 0.54)	-0.11 (-0.54, 0.32)
**Fruits and vegetables**				
<5 months	Ref	Ref	Ref	Ref
5-8 months	-0.49 (-0.72, -0.26)	-0.31 (-0.54, -0.09)	-0.55 (-0.83, -0.27)	-0.34 (-0.61, -0.06)
9-12 months	-0.18 (-0.67, 0.31)	-0.13 (-0.60, 0.33)	0.38 (-0.32, 1.08)	0.47 (-0.17, 1.12)

Exposure	Singletons (n=1150) Unadjusted* Mean difference (95% CI)	Singletons (n=1150) Adjusted† Mean difference (95% CI)	Twins (n=483) Unadjusted* Mean difference (95% CI)	Twins (n=483) Adjusted† Mean difference (95% CI)
**Grains**				
<5 months	Ref	Ref	Ref	
5-8 months	-0.45 (-0.67, -0.24)	-0.26 (-0.47, -0.04)	-0.60 (-0.89, -0.31)	-0.33 (-0.61, -0.05)
9-12 months	-0.35 (-0.90, 0.20)	-0.01 (-0.52, 0.51)	-0.29 (-0.91, 0.34)	-0.19 (-0.77, 0.39)
**Protein**				
5-8 months	Ref	Ref	Ref	Ref
9-12 months	-0.02 (-0.23, 0.20)	0.04 (-0.17, 0.25)	-0.07 (-0.37, 0.23)	-0.12 (-0.39, 0.14)

Ref=Reference group. *Values are mean differences and their 95% confidence intervals from linear mixed models.

†Adjusted for mother's age, race/ethnicity, education, insurance status, smoking, pre-pregnancy BMI, child's gestational age, multiple birth status, Women, Infants, and Children (WIC) participation, and age of juice introduction.

Supplemental Table S4. Childhood anthropometric indicators at 2-3 years of age (n=2492) based on early feeding practices adjusted for age of juice introduction, Upstate KIDS cohort*

	BMI-for-age z score	Weight-for-age z score	Height-for-age z score
**Exposure**	Adjusted† Mean difference (95% CI)		
**Type of feeding**			
Formula feeding	Ref	Ref	Ref
Partial breastfeeding	-0.12 (-0.30, 0.05)	-0.03 (-0.14, 0.09)	0.10 (-0.07, 0.26)
Exclusive breastfeeding	-0.22 (-0.41, -0.04)	-0.13 (-0.26, -0.01)	0.04 (-0.13, 0.22)
**Duration of breastfeeding**			
None (formula feeding)	Ref	Ref	Ref
<6 months	-0.13 (-0.34, 0.07)	-0.07 (-0.19, 0.06)	0.08 (-0.11, 0.27)
6-<12 months	-0.25 (-0.48, -0.02)	-0.07 (-0.22, 0.08)	0.17 (-0.05, 0.39)
≥12 months	-0.42 (-0.65, -0.18)	-0.23 (-0.39, -0.08)	0.15 (-0.08, 0.38)
**Introduction of complementary foods**			
<5 months	Ref	Ref	Ref
5-8 months	-0.02 (-0.18, 0.13)	-0.06 (-0.16, 0.04)	-0.07 (-0.22, 0.08)
9-12 months	0.21 (-0.38, 0.81)	0.04 (-0.33, 0.41)	-0.12 (-0.72, 0.48)
**Dairy**			
<5 months	Ref	Ref	Ref
5-8 months	0.40 (0.12, 0.68)	0.18 (0.00, 0.36)	-0.07 (-0.33, 0.20)
9-12 months	0.40 (0.13, 0.66)	0.09 (-0.07, 0.25)	-0.13 (-0.37, 0.12)
**Fruits and vegetables**			
<5 months	Ref	Ref	Ref
5-8 months	-0.11 (-0.27, 0.05)	-0.06 (-0.16, 0.04)	-0.01 (-0.16, 0.15)
9-12 months	-0.02 (-0.43, 0.38)	-0.02 (-0.26, 0.23)	0.00 (-0.40, 0.40)

	BMI-for-age z score	Weight-for-age z score	Height-for-age z score
**Exposure**	Adjusted† Mean difference (95% CI)		
**Grains**			
<5 months	Ref	Ref	Ref
5-8 months	-0.03 (-0.18, 0.13)	-0.07 (-0.17, 0.03)	-0.07 (-0.22, 0.07)
9-12 months	0.17 (-0.22, 0.57)	0.04 (-0.21, 0.28)	-0.10 (-0.50, 0.30)
**Protein**			
5-8 months	Ref	Ref	Ref
9-12 months	0.11 (-0.04, 0.25)	-0.03 (-0.13, 0.06)	-0.10 (-0.24, 0.03)

Ref=Reference group. *Values are mean differences and their 95% confidence intervals from linear mixed models.

†Adjusted for mother's age, race/ethnicity, education, insurance status, smoking, pre-pregnancy BMI, child's gestational age, multiple birth status, Women, Infants, and Children (WIC) participation, and age of juice introduction.

Supplemental Table S5. Childhood anthropometric indicators at 7-9 years of age (n=1633) based on early feeding practices adjusted for age of juice introduction, Upstate KIDS cohort*

	BMI-for-age z score	Weight-for-age z score	Height-for-age z score
**Exposure**	Adjusted† Mean difference (95% CI)		
**Type of feeding**			
Formula feeding	Ref	Ref	Ref
Partial breastfeeding	-0.08 (-0.29, 0.13)	0.01 (-0.16, 0.17)	0.07 (-0.13, 0.26)
Exclusive breastfeeding	-0.11 (-0.33, 0.11)	0.02 (-0.15, 0.20)	0.10 (-0.10, 0.30)
**Duration of breastfeeding**			
None (formula feeding)	Ref	Ref	Ref
<6 months	-0.29 (-0.52, -0.06)	-0.19 (-0.37, -0.01)	-0.11 (-0.32, 0.10)
6-<12 months	-0.20 (-0.46, 0.07)	-0.01 (-0.22, 0.20)	0.08 (-0.17, 0.33)
≥12 months	-0.28 (-0.56, -0.01)	-0.13 (-0.35, 0.09)	-0.12 (-0.38, 0.14)
**Introduction of complementary foods**			
<5 months	Ref	Ref	Ref
5-8 months	-0.19 (-0.37, -0.01)	-0.05 (-0.20, 0.09)	0.10 (-0.07, 0.27)
9-12 months	0.23 (-0.43, 0.90)	0.17 (-0.38, 0.71)	0.07 (-0.53, 0.68)
**Dairy**			
<5 months	Ref	Ref	Ref
5-8 months	0.30 (-0.01, 0.62)	0.34 (0.10, 0.59)	0.09 (-0.21, 0.38)
9-12 months	0.34 (0.05, 0.63)	0.31 (0.09, 0.53)	0.04 (-0.23, 0.32)
**Fruits and vegetables**			
<5 months	Ref	Ref	Ref
5-8 months	-0.30 (-0.49, -0.10)	-0.18 (-0.34, -0.03)	-0.01 (-0.19, 0.17)
9-12 months	-0.08 (-0.49, 0.34)	-0.14 (-0.49, 0.20)	-0.19 (-0.57, 0.19)
**Grains**			
<5 months	Ref	Ref	Ref
5-8 months	-0.26 (-0.45, -0.08)	-0.06 (-0.21, 0.08)	0.19 (0.02, 0.36)

	BMI-for-age z score	Weight-for-age z score	Height-for-age z score
**Exposure**	Adjusted† Mean difference (95% CI)		
9-12 months	-0.04 (-0.47, 0.39)	-0.05 (-0.40, 0.29)	0.06 (-0.33, 0.46)
**Protein**			
5-8 months	Ref	Ref	Ref
9-12 months	0.03 (-0.14, 0.21)	0.01 (-0.13, 0.15)	-0.01 (-0.18, 0.15)

Ref=Reference group. *Values are mean differences and their 95% confidence intervals from linear mixed models.

†Adjusted for mother's age, race/ethnicity, education, insurance status, smoking, pre-pregnancy BMI, child's gestational age, multiple birth status, Women, Infants, and Children (WIC) participation, and age of juice introduction.
